# Involvement of Glucagon in Preventive Effect of Menthol Against High Fat Diet Induced Obesity in Mice

**DOI:** 10.3389/fphar.2018.01244

**Published:** 2018-11-16

**Authors:** Pragyanshu Khare, Priyanka Mangal, Ritesh K. Baboota, Sneha Jagtap, Vijay Kumar, Dhirendra Pratap Singh, Ravneet K. Boparai, Shyam S. Sharma, Romesh Khardori, Sanjay K. Bhadada, Kanthi K. Kondepudi, Kanwaljit Chopra, Mahendra Bishnoi

**Affiliations:** ^1^National Agri-Food Biotechnology Institute, Sahibzada Ajit Singh Nagar, India; ^2^Department of Pharmacology, University Institute of Pharmaceutical Sciences, Panjab University, Chandigarh, India; ^3^Department of Natural Products, National Institute of Pharmaceutical Education and Research, Sahibzada Ajit Singh Nagar, India; ^4^Department of Biotechnology, Government College for Girls, Chandigarh, India; ^5^Department of Pharmacology and Toxicology, National Institute of Pharmaceutical Education and Research, Sahibzada Ajit Singh Nagar, India; ^6^Division of Endocrinology and Metabolism, The EVMS Sterling Centre of Diabetes and Endocrine Disorders, Department of Internal Medicine, East Virginia Medical School, Norfolk, VA, United States; ^7^Department of Endocrinology, Post Graduate Institute of Medical Education and Research, Chandigarh, India

**Keywords:** obesity, menthol, adipose tissue, glucagon, TRPM8

## Abstract

Glucagon mediated mechanisms have been shown to play clinically significant role in energy expenditure. The present study was designed to understand whether pharmacological mimicking of cold using menthol (TRPM8 modulator) can induce glucagon-mediated energy expenditure to prevent weight gain and related complications. Acute oral and topical administration of TRPM8 agonists (menthol and icilin) increased serum glucagon concentration which was prevented by pre-treatment with AMTB, a TRPM8 blocker. Chronic administration of menthol (50 and 100 mg/kg/day for 12 weeks) to HFD fed animals prevented weight gain, insulin resistance, adipose tissue hypertrophy and triacylglycerol deposition in liver. These effects were not restricted to oral administration, but also observed upon the topical application of menthol (10% w/v). The metabolic alterations caused by menthol in liver and adipose tissue mirrored the known effects of glucagon, such as increased glycogenolysis and gluconeogenesis in the liver, and enhanced thermogenic activity of white and brown adipose tissue. Correlation analysis suggests a strong correlation between glucagon dependent changes and energy expenditure markers. Interestingly, *in-vitro* treatment of the serum of menthol treated mice increased energy expenditure markers in mature 3T3L1 adipocytes, which was prevented in the presence of non-competitive glucagon receptor antagonist, L-168,049, indicating that menthol-induced increase in serum glucagon is responsible for increase in energy expenditure phenotype. In conclusion, the present work provides evidence that glucagon plays an important role in the preventive effect of menthol against HFD-induced weight gain and related complications.

## Introduction

Glucagon, a 29 amino acid insulin counter-regulatory peptide hormone, is secreted by α-cells in the pancreas. The increase in glucagon levels in blood, primarily due to hypoglycaemia, results in synthesis, and activation of enzymes responsible for gluconeogenesis and glycolysis in hepatocytes (Elrick, 1956). It increases hepatic glucose output and increase energy mobilization *via* glucose utilization (Habegger et al., 2010; Heppner et al., 2010; Ramnanan et al., 2011; Müller et al., 2017). Glucagon has wide range of actions outside its actions on glucose homeostasis, that is lowering of blood cholesterol levels, increase in fatty acid catabolism, hepatic fibroblast growth factor-21 (FGF-21) production, suppressive effects on hunger and food intake, thermogenesis, and enhanced energy expenditure (Habegger et al., 2010; Heppner et al., 2010; Müller et al., 2017). All these actions are of significance for positive energy expenditure state and hence for obesity prevention and therapeutics. There are different mechanisms of action of glucagon induced energy expenditure including enhanced activation of brown adipose tissue (BAT) (Billington et al., 1991; Kinoshita et al., 2014). Uncoupling protein-1 (UCP-1)-positive BAT in adult humans (supraclavicular neck region) and rodents can be activated by a variety of stimuli including cold exposure (Sacks and Symonds, 2013; Brychta and Chen, 2017; Mo et al., 2017). Glucagon knockout mice have reduced thermogenic responses to cold exposure and pharmacological adrenergic stimulation, which is restored by glucagon replacement (Kinoshita et al., 2014). Hence, glucagon is an important mediator of cold exposure induced thermogenesis/energy expenditure. Through this manuscript, we focus on the observed link between cold exposure and glucagon release that has not fully explored in this perspective (Kuroshima and Doi, 1976; Kuroshima et al., 1981; Doi et al., 1982; Guezennec et al., 1988; Habegger et al., 2010; Heppner et al., 2010) through pharmacological activation of cold sensing receptor, Transient Receptor Potential cation channel subfamily Melastatin member 8 (TRPM8), also known as the cold and menthol receptor 1 (CMR1).

TRPM8 is a cold-receptor and can sense non-noxious cold temperatures i.e., 18–25°C (McKemy et al., 2002; Peier et al., 2002; Bautista et al., 2007). TRPM8 knock-out mice display a defective response to cooling agents and cold stimuli (Colburn et al., 2007; Dhaka et al., 2007), which indicates that this cold-sensing channel plays a physiologically relevant role in the detection of environmental temperature in mammals. TRPM8 is highly expressed in subsets of sensory neurons i.e., sensory nerve endings innervating the skin and gut (Dhaka et al., 2008; Harrington et al., 2011; Bidaux et al., 2015; De Jong et al., 2015). TRPM8 is functionally expressed in rodent white adipose tissue (WAT; Jiang et al., 2017) and BAT (Ma et al., 2012) as well as human WAT (Rossato et al., 2014). Menthol administration (topically 5% for 3 or 9 days) produces a persistent increase in energy expenditure without affecting food intake (Vizin et al., 2018). Tajino and colleagues have also reported that topical menthol application led to an increase in TRPM8 dependent core body temperature, which was positively correlated with *UCP1* expression in BAT (Tajino et al., 2007). TRPM8-deficient mice, housed in a mild cold environment, displayed an increase in tail heat loss and lower core body temperature, associated with lipid metabolic dysfunction and late onset of obesity (Reimúndez et al., 2018). TRPM8 is also involved in the priming of mitochondria to perform uncoupled respiration (Goralczyk et al., 2017). Activation of TRPM8 on human white adipocytes by menthol and icilin induced a rise in intracellular calcium and *UCP1* expression, increased mitochondrial membrane potential, glucose uptake and heat production (Goralczyk et al., 2017). The effect was predominant in white adipocytes (*browning*) than in brown adipocytes (Goralczyk et al., 2017). Report suggests that genetic polymorphisms of TRPM8 genes may modify individual susceptibility to metabolic syndrome (Tabur et al., 2015).

The present manuscript is an attempt to uncover additional mechanisms linking TRPM8 and energy expenditure, i.e., involvement of glucagon. We sought to understand the link between pharmacological mimicking of cold sensation, glucagon machinery and induction of “*brite*” phenotype in WAT. Briefly, our data strongly indicate that pharmacological activation of cold receptor TRPM8 by menthol plays an important role in energy homeostasis *via* activation of glucagon machinery which provides an additional mechanism for TRPM8 activation induced prevention of obesity and related conditions.

## Materials and methods

### Reagents and material

Menthol (PubChem CID: 16666), icilin (PubChem CID: 161930), N-(3-Aminopropyl)−2-[(3-methylphenyl) methoxy] -N-(2-thienylmethyl) benzamide hydrochloride (AMTB, PubChem CID: 16095383) and D-glucose were purchased from Sigma Aldrich (Sigma Aldrich, St. Louis, MO, USA). Isopropyl alcohol, trizol reagent, fetal bovine serum (FBS), and penicillin/streptomycin (P/S) were purchased from invitrogen (Invitrogen, Camarillo, CA, USA). Mouse 3T3-L1 preadipocyte (3T3-L1 PA), Dulbecco's modified Eagle's medium (DMEM), maintenance media (MM) and differentiation medium (DM) were purchased from Zenbio (Zenbio Inc. Research Triangle Park, NC). L-168,049 (4-[3-(5-Bromo-2-propoxyphenyl)-5-(4-chlorophenyl)-1H-pyrrol-2-yl] pyridine; PubChem CID: 5311276) was purchased from Tocris Bioscience (Tocris Bioscience, Bristol, UK).

**Chemical compounds studied in this article**-Menthol; Icilin; AMTB hydrochloride; L-168,049

### Animal experiments

#### Animals

Male Swiss albino mice (7–8 week old; 18–22 g) were obtained from and housed in the Central Animal Facility, National Institute of Pharmaceutical Education and Research (NIPER), S.A.S. Nagar, Punjab, India. Animals were maintained under standard laboratory conditions (temperature 22 ± 2°C; humidity 55 ± 5%; and 12 h light/dark cycle) and provided with diets and water *ad libitum*. This study was carried out in accordance with the recommendations of “Committee for the Purpose of Control and Supervision of Experiments on Animals (CPCSEA)” guidelines on the uses and care of experimental animals. The protocols used in this study were approved by “Institutional Animal Ethics Committee (IAEC) of NIPER.” After procurement, mice were acclimatized to experimental conditions for one week followed by weight based randomization of animals for each experiment separately.

#### Acute studies

All the acute studies were done in 6 h fasted animals. Following experiments were performed during acute studies. Doses and route of administration of agents were determined on the bases of previous literature (Colburn et al., 2007; Lashinger et al., 2008; Plevkova et al., 2013; Uvin et al., 2015).

**Experiment 1A:** To study the effect of acute oral administration of TRPM8 agonists, menthol and icilin, on serum glucagon levels and core body (rectal) Temperature in Mice

**Experiment 1B:** To study the effect of pre-treatment of TRPM8 antagonist, AMTB on orally administered menthol induced increase in serum glucagon levels and core body (rectal) temperature in Mice

Animals were divided in 4 groups of 6–8 animals/group. Group 1, 2, 3, 4 were administered vehicle, menthol (200 mg/kg, *p.o*.), icilin (20 mg/kg *p.o*.), and pre-treatment of AMTB (4 mg/kg *i.p*.) followed by menthol (200 mg/kg) respectively. In groups 1, 2, 3 rectal temperature was measured upto 120 min and serum glucagon levels were measured at 120 min. In pre-treatment group, AMTB was administered 30 min prior to menthol administration and rectal temperature measurement was done for next 120 min and serum glucagon levels were measured at 120 min after the menthol administration.

**Experiment 2A:** The effect Of acute topical application of Trpm8 agonists, menthol and icilin, on serum glucagon levels, and core body rectal temperature in mice

**Experiment 2B:** The effect of Pre-treatment of TRPM8 antagonist, AMTB on topically applied menthol induced increase in serum glucagon levels, and core body rectal temperature in mice

Animals were divided in 4 groups of 6–8 animals/group. Group 1, 2, 3, 4 were topically applied vehicle, menthol (10 % w/v), icilin (1 % w/v), and pre-treatment of AMTB (1 % w/v) followed by menthol (10 % w/v) respectively in the selected region of abdominal area. In groups 1, 2, 3 rectal temperatures were measured for 120 min and serum glucagon levels were measured at 120 min. In pre-treatment group, AMTB was administered 30 min prior to menthol administration and rectal temperature measurement was done for next 120 min and serum glucagon levels were measured at 120 min after the menthol application.

Doses of menthol and icilin were prepared in 0.2% Tween-80 using traditional pharmaceutical technique. Briefly, weighed amount of chemical was carefully triturated with required amount of tween 80 and then remaining solvent was mixed step wise with continuous trituration. AMTB was dissolved in 0.9% saline water for *i.p*. dose. 0.2% tween 80 solution was administered/applied as vehicle.

#### Chronic high fat diet (HFD)-induced obesity studies:

**Experiment 3:** The effect of chronic oral and topical co-administration of menthol against HFD-induced weight gain and associated complications

Animals were divided in 6 groups of 6–8 animals/group. Group 1, 2, 3, 4, 5, 6 were administered NC (Normal rodent chow), HFD (60 % energy by fat), menthol (50 mg/kg *p.o*.) with HFD, menthol (100 mg/kg *p.o*.) with HFD, topical menthol (10 % w/v) with HFD, and menthol (100 mg/kg *p.o*.) with NC, respectively. 0.2% tween 80 was administered as vehicle in NC and HFD fed mice. All the animals were given respective treatments for 12 weeks with weekly measurement of body weight. During 13th week, core body rectal temperature and oral glucose tolerance test was performed (see details below, in methods). On the day of sacrifice, animals were fasted for 6 h and blood was collected for different biochemical tests and ELISA including glucagon levels (see details in methods section). Animals were sacrificed and tissue (visceral WAT (vWAT), BAT, liver, hypothalamus) were collected, processed, and stored at −80°C for further biochemical, gene expression immunohistochemistry and histological analysis (see details in methods section).

### Methods

#### Composition of HFD

For diet induced obesity experiments high fat diet (HFD, 60% calories of food derived from fat) was prepared in-house by earlier reported method (Srinivasan et al., 2005), with slight modifications. The composition of HFD was, NC (36.5% w/w; Ashirwad-Industries, Chandigarh); Milk casein (25.0% w/w; Spectrochem Pvt.Ltd, Mumbai, India); Animal lard (32.0% w/w; Local market, Chandigarh, India); Vitamin and mineral mix (6.0% w/w); Yeast extract (0.1% w/w); dl methionine (0.3% w/w, Himedia laboratories Pvt.Ltd, Mumbai, India); Sodium chloride (0.1% w/w; Himedia laboratories Pvt.Ltd, Mumbai, India).

#### Rectal temperature measurement

To assess core body rectal temperature (as a marker of cold induced energy expenditure (Abreu-Vieira et al., 2015), rectal temperature was measured using rectal probe assisted temperature monitoring device (Homeothermic Blanket Control Unit, Harvard Apparatus, Holliston, MA). Briefly, 3 cm of mouse specific probe was inserted into mouse anus, after 30 s stabilization period display reading was noted. All the measurements at single time point were repeated 3 times.

#### Morphometric parameters

Naso-anal length and circumference of lower abdomen was measured using length measuring tape according to the earlier described method (Novelli et al., 2007) and Lee's index (weight^0.33^ × 1000)/naso-anal length; (Bernardis, 1970; Novelli et al., 2007) was calculated.

#### Oral glucose tolerance test

During the last week of chronic experiment, oral glucose tolerance test (OGTT) was performed. On the day of experiment mice were fasted for 6 h. Blood glucose concentration was measured after 15, 30, 45, 60, 90, and 120 min of oral administration of 2 g/kg of D-glucose using Glucocard^TM^ (Arkray Factory Inc., Shiga, Japan), Area under the concentration-time curve (AUC) was calculated using graph pad prism 6 software.

#### Analysis of blood biochemical parameters

Blood was allowed to coagulate for 20 min on ice followed by centrifugation at 2,500 g for 15 min at 4°C to obtain serum. Serum triglyceride, glucose (Accurex Biomedical Pvt. Ltd., Mumbai, India), and free fatty acids (FFA) were estimated by colorimetric assay. Glucagon, insulin (Ray Biotech, Norcross, GA, USA), leptin, adiponectin (Invitrogen, Camarillo, CA, USA), FGF21 and pAMPK (Cusabiotech, Wuhan, China) levels in serum and different tissues were determined using ELISA as per the manufacturer's instructions.

#### Adipose tissue metal analysis

vWAT and BAT were weighted. Further, these were microwave digested using HNO3 (Suprapur® Merck, Germany). These digested samples were used for metal analysis by using Inductively Coupled Plasma Mass Spectrometry (ICP-MS; 7700 × Agilent Technologies, Santa Clara, CA; Wang et al., 2017).

#### Estimation of liver triglycerides

Liver lipids were estimated using classical phase separation based method (Folch et al., 1957). Briefly fixed amount of liver tissue was homogenized into phosphate buffer and supernatant was partitioned into mixture of chloroform: methanol (3:1); lower layer was evaporated using nitrogen flushing. Remnant was dissolved in 2.0 % Triton X to estimate triglyceride content.

#### Estimation of liver glycogen

Liver glycogen was estimated using classical iodine method (Van der Vies, 1954). Briefly liver glycogen was extracted using 5% trichloroacetic acid. 2 parts clear filtrate was treated with 3 parts of iodine reagent and absorbance was measured at 650 nm using a micro plate reader (Spectra max M5^e^, Molecular Devices, Minnesota, USA). Iodine reagent was prepared by dissolving 16.5 ml Lugol's solution (1 g iodine and 2 g of KI in 20 ml water) to 990 ml of 25% KCl solution.

#### RNA extraction and qPCR

Total RNA from vWAT, BAT, and liver was extracted using Trizol based RNA extraction method described elsewhere (Khare et al., 2016). Briefly, tissues were homogenized in Trizol followed by the phase separation using chloroform. Total RNA was pelleted down by isopropanol assisted RNA precipitation. Total RNA was checked for integrity on 1.2% agarose gel and quantified by Infinite® M200 Pro NanoQuant at 260/280 nm (Tecan, Switzerland). RT-first strand synthesis kit (Qiagen, CA, USA) was used to reverse transcribed 2.0 μg of total RNA into cDNA as per the manufacturer's instructions. Changes in gene expression relative to control were determined by qPCR (Applied Biosystems 7500 Fast Real-Time PCR) using SYBR green in the following conditions: 95°C for 10 min, followed by 40 cycles of 95 and 60°C for 1 min. ΔΔC_t_ method was used to analyze qPCR data, values were expressed in terms of fold change (FC) relative to the control group. Sequences of primers are presented in Table 1.

**Table 1:** Sequence of primers used in this study.

**Primer**	**Forward**	**Reverse**
Glucagon receptor	CCTCTGCCCAGGTAATGGA	TAGGTACCAGGGGCAGGAA
TRPM8	GGAGCCGCAGAAATGGTACT	GCTCTGGGCATAACCACACT
Cox5a	CGCTGTCTGTTCCATTCGCT	CGTCTACATGCTCGCAATGC
ERR α	TTCCAGGGCACAAGGAGGA	ACA GCT GTA CTC GAT GCT CC
TMEM 26	CCCTACTCTGGTCTCTGGCA	GGAAGGGACCGTCTTGGATG
CD137	GCTCCTCTACCCACAACGC	GTTCGTCCAGGGTCGACAG
Tfam	TCGCATCCCCTCGTCTATCA	CCACAGGGCTGCAATTTTCC
COX 7a	CAGGCTCTGGTCCGGTCT	CCCCCAGAGTCAGCGTCA
Idh3a	AGGGAAGTTGCGGAGAACTG	TGG CAA CAC CGT TGG CTC
ATP5b	GTCCCGGGCAAGAAAGATACA	ACGGCTTCTTCAATGGGTCC
β actin	TGGTGGGAATGGGTCAGAAG	ACGGTTGGCCTTAGG GTTCA
G6Pase	GTGCAGCTGAACGTCTGTCTGT	TCCGGAGGCTGGCATTGT
PEPCK	GAACTGACAGACTCGCCCTATGT	GTTGCAGGCCCAGTTGTTG
Glucokinase	CGGATGGTGGATGAGAGCTC	CATTTCACAGGGCAGGGGAT
GYS-2	AGTCCTCAGTGCAGTGATGC	CTGCATCAGGGTGTGGACTT
PPAR-γ	GCCAAGGTGCTCCAGAAGAT	CTTTGTCAGCGACTGGGACT
C/EBP α	CCAGTGACAATGACCGCCT	CGACCCTAAACCATCCTCCG
UCP1	Customized RT^2^ Profiler PCR Array SABiosciences kits Catalogue number-CAPM 12378	
PGC1α		
PRDM16		
CIDEA		
BMP4		
FOXC2		
TBX1		

#### Adipose tissue histology and immunohistochemistry

Adipose tissue was *in situ* fixed by 10% v/v buffered formalin then isolated and stored in 10% buffered formalin for 48 h. Small pieces of adipose tissue then serially dehydrated using gradient ethanol, then embedded in paraffin. Ten microgram section of adipose tissue were made and stained using Haematoxylin and Eosin staining (H&E) and all the images were captured using 20X objective (Final camera magnification 14X). The mean adipocytes size in adipose tissue sections were estimated in 5 different tissue images using Imagescope (Version 12.1.0.5029, Aperio Technologies Inc.) by manual counting.

For immunohistochemistry, tissue sections were deparaffinised followed by antigen retrival using citrate buffer method. Five percent v/v goat serum was used to prevent nonspecific antibody binding. Slides were incubated overnight with rabbit poly clonal anti-*UCP1* primary antibody (1:50; Thermo Scientific Pierce Antibodies, Rockford, IL, US) followed by incubation with FITC leveled goat anti-rabbit secondary antibody (1; 2500; Thermo Scientific Pierce Antibodies, Rockford, IL, US) for 2 h at room temperature. DAPI was used to counter stain the nucleus of the cells.

#### 3T3-L1 cell culture

3T3-L1 preadipocytes (Passage no-20, Population Doubling Time-1day) were maintained in DMEM supplemented with FBS (10% v/v) and antibiotics (P/S (1 % v/v) until confluence. Differentiation of 3T3-L1 preadipocytes was induced by replacing DMEM with differentiation media for two days. Then, differentiation media was replaced by maintenance media for next 8–10 days with media replacement on every second day. On maturation, adipocytes were treated with maintenance media in which FBS was replaced with serum collected from vehicle and menthol treated animals. mRNA expression analysis of energy expenditure genes at different time points of treatment (i.e., 4, 8, and 16 h post treatment) was carried out. The effective treatment schedule was again repeated in the presence of L-168,049, a non-competitive glucagon receptor antagonist, at reported effective dose (0.3 μM) (Cascieri et al., 1999).

#### Statistical analysis

All the values are expressed as mean ± SEM. Group comparison was done using One way/Repeated measure ANOVA followed by Tukey's *post-hoc* test. A Pearson correlation analysis was performed using Graph Pad Prism 6 Software (Graph Pad Software Inc., La Jolla, CA, USA). *P* ≤ 0.05 was considered statistically significant.

## Results

### Acute oral administration and topical application of TRPM8 agonists (menthol and icilin) induced serum glucagon and rectal temperature increase in mice

**Figure 1:**
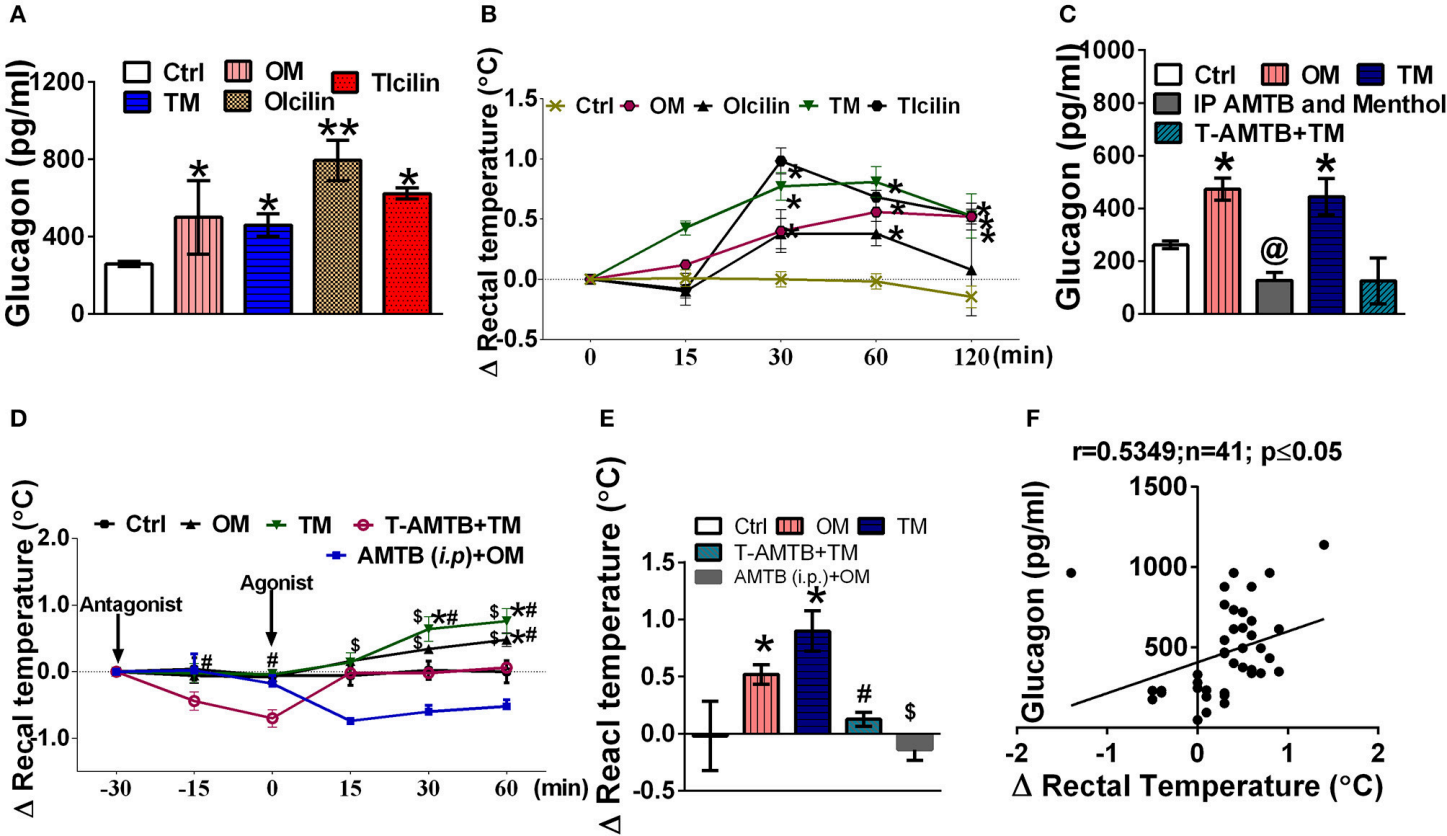
TRPM8 activation increased serum glucagon level and rectal temperature. Effect of oral and topical administration of menthol and icilin on **(A)** serum glucagon levels after 120 min of the treatment; **(B)** change in rectal temperature for 120 min of the treatment; **(C)** serum glucagon levels after 120 min in presence and absence of AMTB pre-treatment; **(D)** change in rectal temperature for 90 min in presence and absence of AMTB pre-treatment, menthol (*p.o*.), and menthol topical was administered as a reference, **(E)** change in rectal temperature after 120 min of the menthol treatment in presence and absence of AMTB pre-treatment, and **(F)** correlation between change in rectal temperature and serum glucagon levels after 120 min of the treatment. Ctrl, vehicle treated animal; OM, menthol (per oral; 200 mg/kg); TM, menthol (10% topical application); OIcilin, icilin (*p.o*.; 20 mg/kg); TIcilin, icilin (1% topical application); T-AMTB+TM, AMTB (1% topical application) followed by menthol (10% topical application) 30 min after AMTB; IP AMTB and menthol, AMTB (4 mg/kg *i.p*.) followed by Menthol (200 mg/kg, *p.o*.) 30 min after AMTB. *n* = 5. All the values are expressed as mean ± S.E.M; ^**^p < 0.01 vs. Ctrl group, ^*^p < 0.05 vs. Ctrl group, ^@^*p* < 0.05 vs. OM, ^$^*p* < 0.05 vs. AMTB (*i.p*.)+OM, and ^#^*p* < 0.05 vs. T-AMTB+TM analyzed by one way ANOVA **(A,C,E)** or Repeated measure (RM) ANOVA **(B,D)** followed by Tukey's multiple comparison; Spearman correlation analysis was performed in **(F)**.

TRPM8 agonists, menthol (200 mg/kg) and icilin (20 mg/kg) elevated serum glucagon levels after 2h of administration (Figure 1A). Simultaneously, they also increased rectal temperature [marker of adaptive thermogenesis induced energy expenditure (Lowell and Spiegelman, 2000; Abreu-Vieira et al., 2015)] by ~0.5°C (Figure 1B). Pre-blocking of TRPM8 using AMTB, a TRPM8 blocker prevented oral menthol induced rise in serum glucagon levels and rectal temperature (Figures 1C–E). Also, topical application of TRPM8 agonists, menthol (10 % w/v) and icilin (1 % w/v) elevated serum glucagon levels after 2 h of administration (Figure 1A). Simultaneously, they also increased rectal temperature (Figure 1B). Pre-blocking of TRPM8 using AMTB prevented topically menthol induced rise in serum glucagon levels and rectal temperature (Figures 1C–E). At 2 h, the increase in serum glucagon and rectal temperature were positively correlated in case of oral administration and topical application of menthol and icilin (Figure 1F).

### Chronic oral administration and topical application of menthol prevents HFD-induced weight gain, biochemical, and morphometric alterations in mice

**Figure 2:**
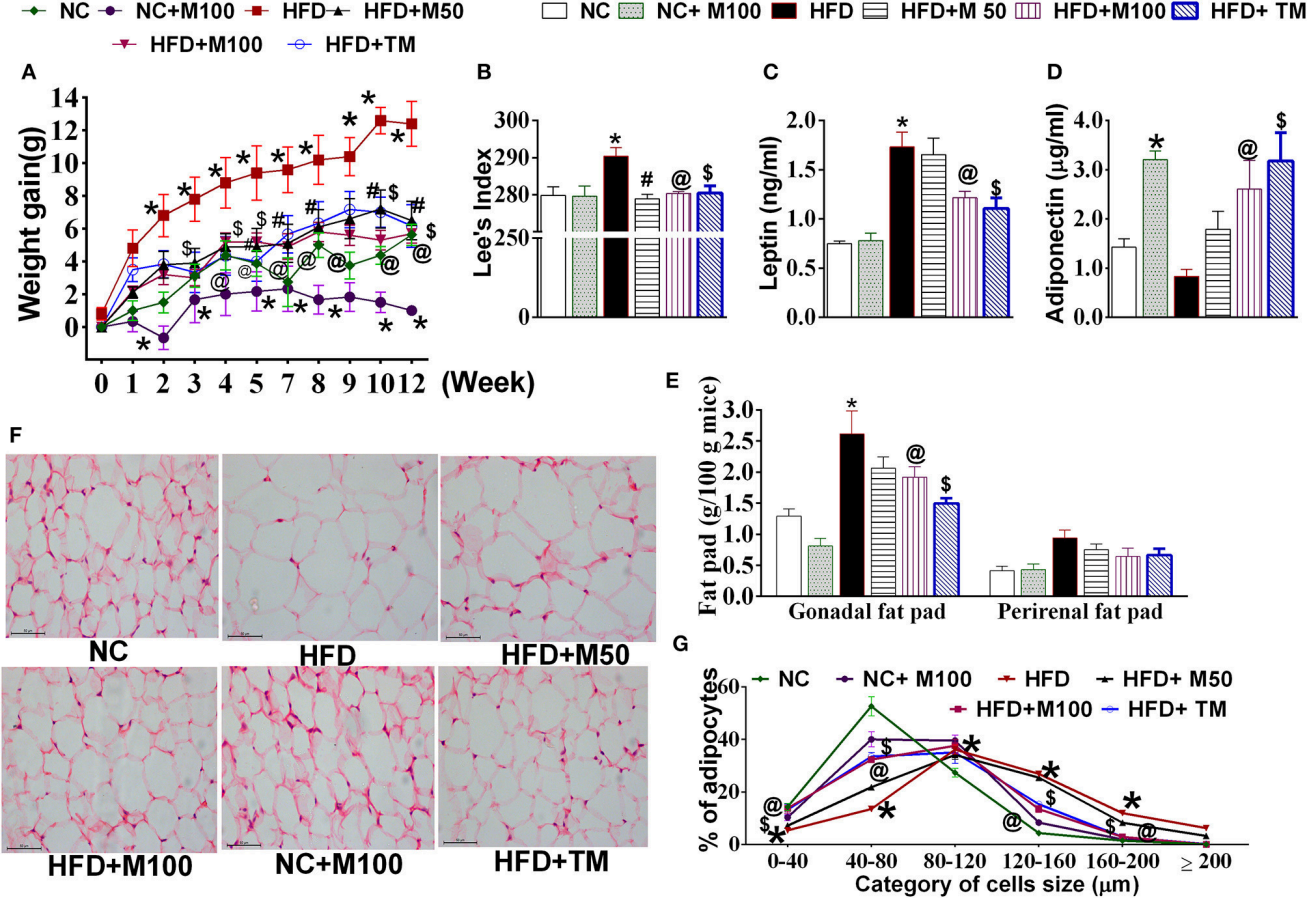
Effect of chronic oral and topical administration of menthol against HFD feeding in mice. **(A)** Weekly change in body weight; **(B)** Lees's index; **(C)** serum leptin levels (using ELISA); **(D)** serum adiponectin levels (using ELISA); **(E)** visceral adipose tissue weight; **(F)** representative image of H&E stained sections of mouse gonadal fat tissue; and **(G)** frequency distribution analysis of adipocyte cell size in H&E stained sections of mouse gonadal fat tissue. NC, normal rodent chow fed; HFD, high fat diet fed; HFD+M50, HFD fed plus daily administration of 50 mg/kg menthol (*p.o*.); HFD+M100, HFD fed plus daily administration of 100 mg/kg menthol (*p.o*.); NC+M100, NC fed plus daily administration of 100 mg/kg menthol (*p.o*.); HFD+TM, HFD fed plus daily application of 10% topical menthol. *n* = 5. All the values are expressed as mean ± S.E.M; Analysis was carried out by RM ANOVA **(A,G)** or by one way ANOVA followed by Tukey's multiple comparison **(B–E)**; ^*^p < 0.05 HFD and NC+M100 vs. NC; ^#^*p* < 0.05 HFD+M50 vs. HFD fed group; ^@^*p* < 0.05 HFD+M100 vs. HFD fed group; ^$^*p* < 0.05 TM vs. HFD fed group.

Co-administration of HFD plus oral menthol (50 and 100 mg/kg) for 12 weeks significantly prevented HFD induced weight gain (Figure 2A) and reversed the altered morphometric and biochemical parameters i.e., Lee index, serum leptin, and adiponectin levels (Figures 2B–D) associated with weight gain. Remarkably, menthol treatment for 12 weeks also prevented weight gain in animals on NC diet (*per se* effect) (Figure 2A). HFD feeding increased percent visceral adipose tissue weight and adipocytes size as compared to NC fed mouse (Figures 2E,F). HFD-induced increased percent visceral adipose tissue weight along with increased adipocytes size (Figures 2E,F) was prevented by chronic menthol co-administration in HFD fed mouse. Similar to NC diet fed mouse the frequency distribution of larger adipocytes were less in menthol co-administered animals whereas number of smaller adipocytes were significantly high as compared to HFD fed mouse (Figure 2G) On the similar ground, chronic daily topical menthol (10 % w/v) application on abdomen of HFD fed mouse for 12 weeks significantly prevented HFD induced weight gain and Lee's index, altered adiponectin, leptin levels (Figures 2A–D), reduced visceral adipose tissue mass, and prevented adipocyte hypertrophy (Figures 2E–G).

### Chronic menthol administration activates glucagon machinery in liver and prevents HFD induced insulin resistance and ectopic fat deposition in liver

**Figure 3:**
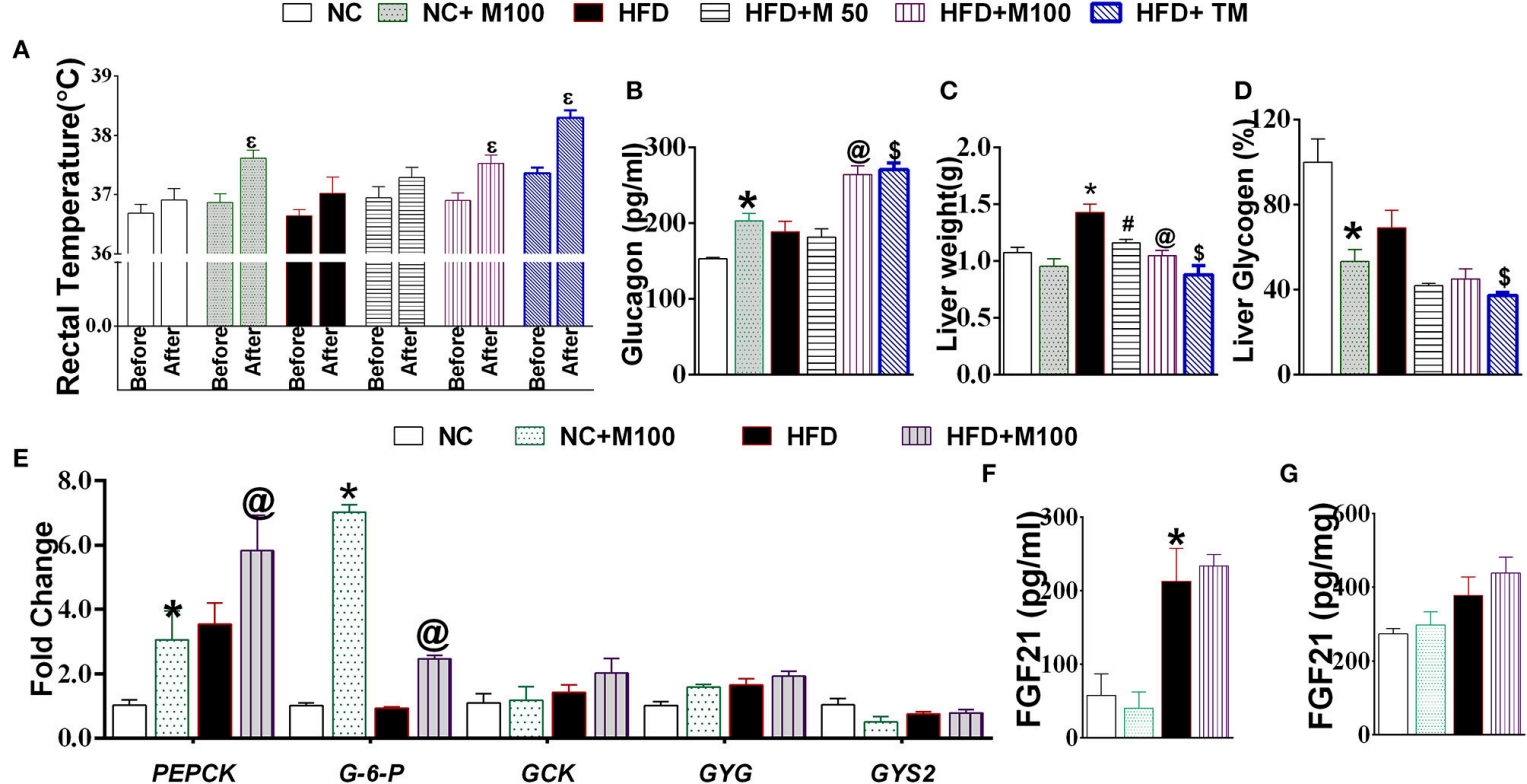
Effect of chronic administration of menthol on glucagon dependent machinery in liver. **(A)** Rectal temperature before and 2 h after menthol treatment, *n* = 5; **(B)** Serum glucagon, *n* = 5; **(C)** Liver weight, *n* = 5; **(D)** Liver glycogen, *n* = 5; **(E)** Expression of gluconeogenic marker genes in liver, *n* = 3; **(F)** Serum FGF21, *n* = 4; **(G)** Liver FGF21, *n* = 4. NC, normal rodent chow fed; HFD, high fat diet fed; HFD+M50, HFD fed plus daily administration of 50 mg/kg menthol (*p.o*.); HFD+M100, HFD fed plus daily administration of 100 mg/kg menthol (*p.o*.); NC+M100, NC fed plus daily administration of 100 mg/kg menthol (*p.o*.); HFD+TM, HFD fed plus daily application of 10% topical menthol. All the values are expressed as mean ± S.E.M; Analysis was carried out by one way ANOVA followed by Tukey's multiple comparison; ^E^*p* < 0.05 rectal temperature before treatment vs. after treatment; ^*^p < 0.05 HFD and NC+M100 vs. NC; ^#^*p* < 0.05 HFD+M50 vs. HFD fed group; ^@^*p* < 0.05 HFD+M100 vs. HFD fed group; ^$^*p* < 0.05 TM vs. HFD fed group.

Menthol administration at 100 mg/kg dose and topical application (10% v/v) increased core body rectal temperature in mouse (Figure 3A). Chronic co-administration of oral and topical menthol in HFD fed mice increased serum glucagon levels (Figure 3B) and decreased liver weight and liver glycogen content (Figures 3C,D). Liver mRNA expression of phosphoenolpyruvate carboxykinase (*PEPCK)* and glucose 6 phosphatase (*G-6pase*) increased significantly after menthol administration in both HFD as well as NC fed mice (Figure 3E). Significant effect was not observed in glucokinase, glycogenin, and glycogen synthase (*GSY2*) expression (Figure 3E). There was non-significant increase in FGF-21 levels in serum and liver (Figures 3F,G).

**Figure 4:**
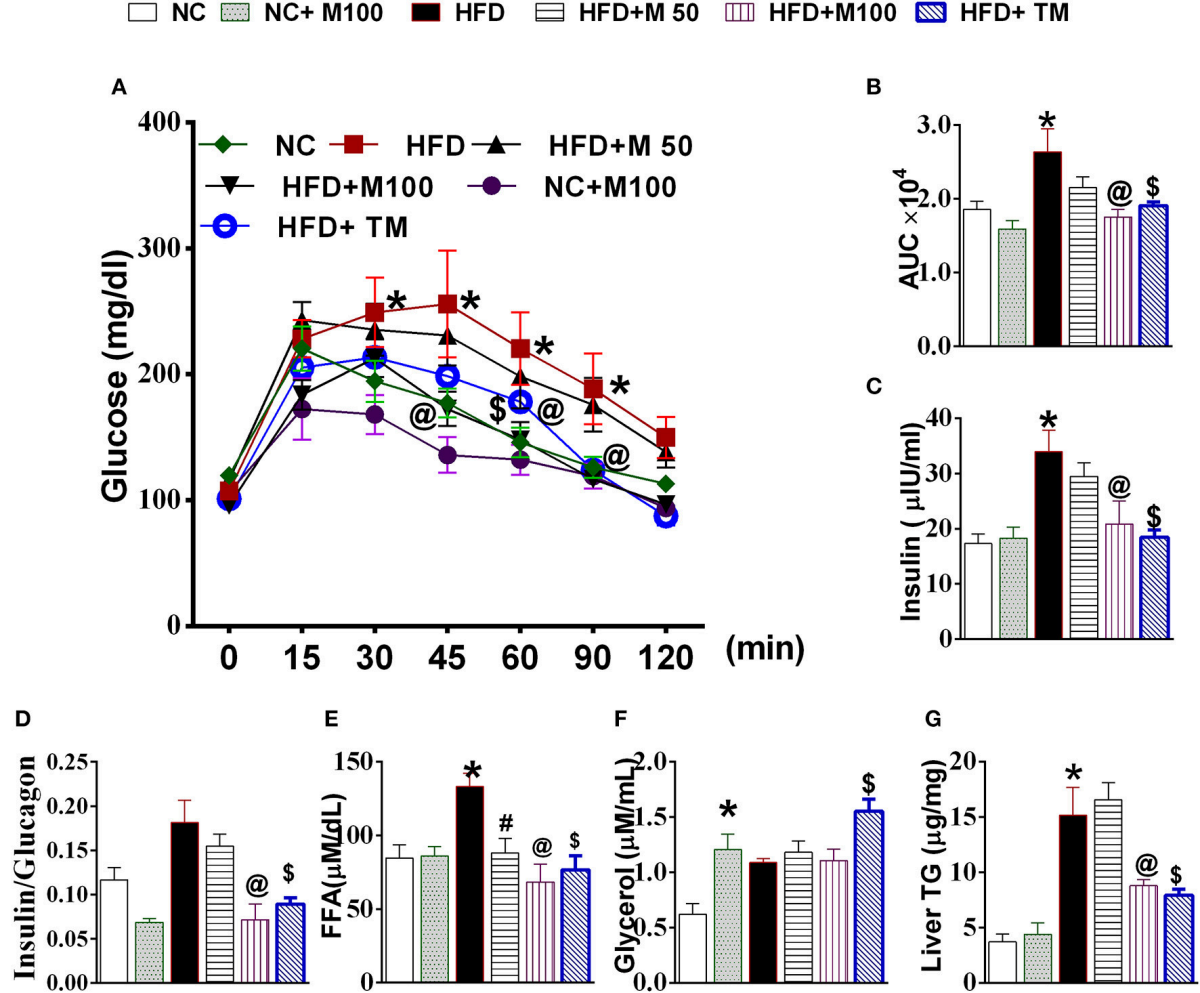
Effect of chronic administration of oral and topical menthol on glucose clearance, hepatic fat deposition, and related parameters. **(A)** oral glucose tolerance test; **(B)** Area under the concentration-time curve (AUC) during OGTT test; **(C)** fasting serum insulin (ELISA); **(D)** insulin:glucagon ratio; **(E)** serum FFA; **(F)** serum glycerol; **(G)** liver triglyceride. NC, normal rodent chow fed; HFD, high fat diet fed; HFD+M50, HFD fed plus daily administration of 50 mg/kg menthol (*p.o*.); HFD+M100, HFD fed plus daily administration of 100 mg/kg menthol (*p.o*.); NC+M100, NC fed plus daily administration of 100 mg/kg menthol (*p.o*.); HFD+TM, HFD fed plus daily application of 10% topical menthol. *n* = 5; All the values are expressed as mean ± S.E.M; Analysis was carried out by one way ANOVA followed by Tukey's multiple comparison; ^*^p < 0.05 HFD and NC+M100 vs. NC; #*p* < 0.05 HFD+M50 vs. HFD fed group; ^@^*p* < 0.05 HFD+M100 vs. HFD fed group; ^$^*p* < 0.05 TM vs. HFD fed group.

HFD fed animals display impaired glucose clearance in OGTT, high serum insulin, serum free fatty acids and high liver TG levels (Figures 4A–C,E,G). Both oral and topical menthol treatment for 12 weeks reversed the HFD induced impaired glucose clearance, ectopic fat deposition in liver, increased insulin and FFA levels in serum (Figures 4A–C,E,G). Interestingly, both oral and topical menthol treatment significantly decreased insulin: glucagon ratio (catabolism marker) and increased serum glycerol levels (Figures 4D,F).

### Chronic menthol administration enhances energy expenditure makers in adipose tissue

**Figure 5:**
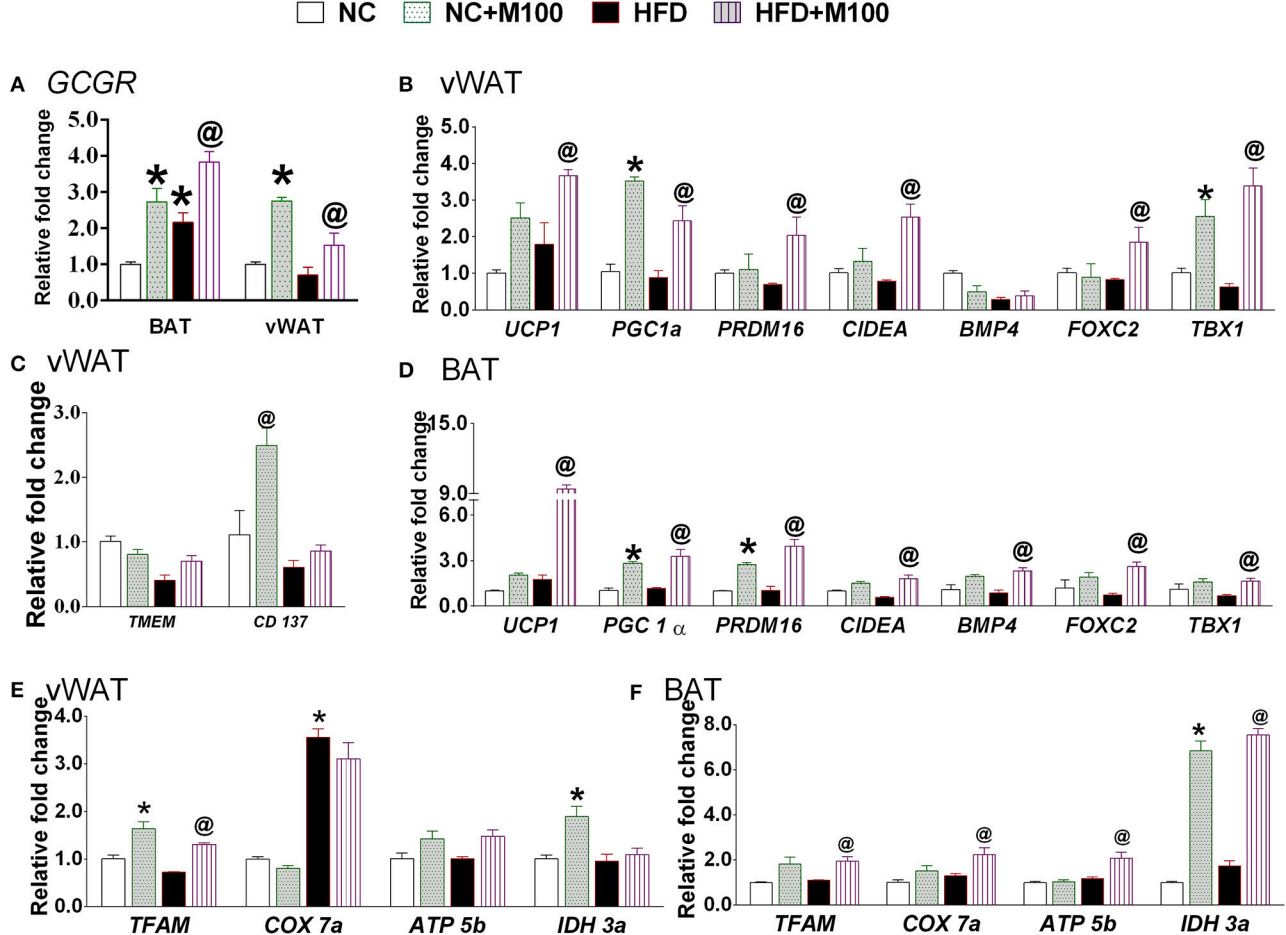
Effect of chronic administration of menthol in energy expenditure related parameters in adipose tissue. **(A)** Glucagon receptor (GCGR) in vWAT and BAT; **(B)** energy expenditure genes in vWAT; **(C)** beige cell marker genes in vWAT; **(D)** energy expenditure genes in BAT; **(E)** mitochondrial activity marker gene in vWAT; **(F)** mitochondrial activity marker gene in BAT. NC, normal rodent chow fed; HFD, high fat diet fed; HFD+M100, HFD fed plus daily administration of 100 mg/kg menthol (*p.o*.); NC+M100, NC fed plus daily administration of 100 mg/kg menthol (*p.o*.). *n* = 3; All the values are expressed as mean ± S.E.M; Analysis was carried out by one way ANOVA followed by Tukey's multiple comparison; ^*^p < 0.05 HFD and NC+M100 vs. NC; ^@^*p* < 0.05 HFD+M100 vs. HFD fed group.

The mRNA expression of glucagon receptor was found to be significantly increased after chronic menthol administration at its effective dose in BAT and vWAT (Figure 5A). Further, chronic menthol administration significantly increased mRNA expression of *browning* markers i.e. Uncoupling Protein 1 (*UCP 1*), Peroxisome proliferator-activated receptor gamma coactivator 1-alpha (*PGC-1*α), PR domain containing 16 (*PRDM16*), Cell death-inducing DFFA-like effector A (*CIDA*), Forkhead box protein C2 (*FOXC2*) and *TBX1* in vWAT and BAT (Figures 5B,D).

**Figure 6:**
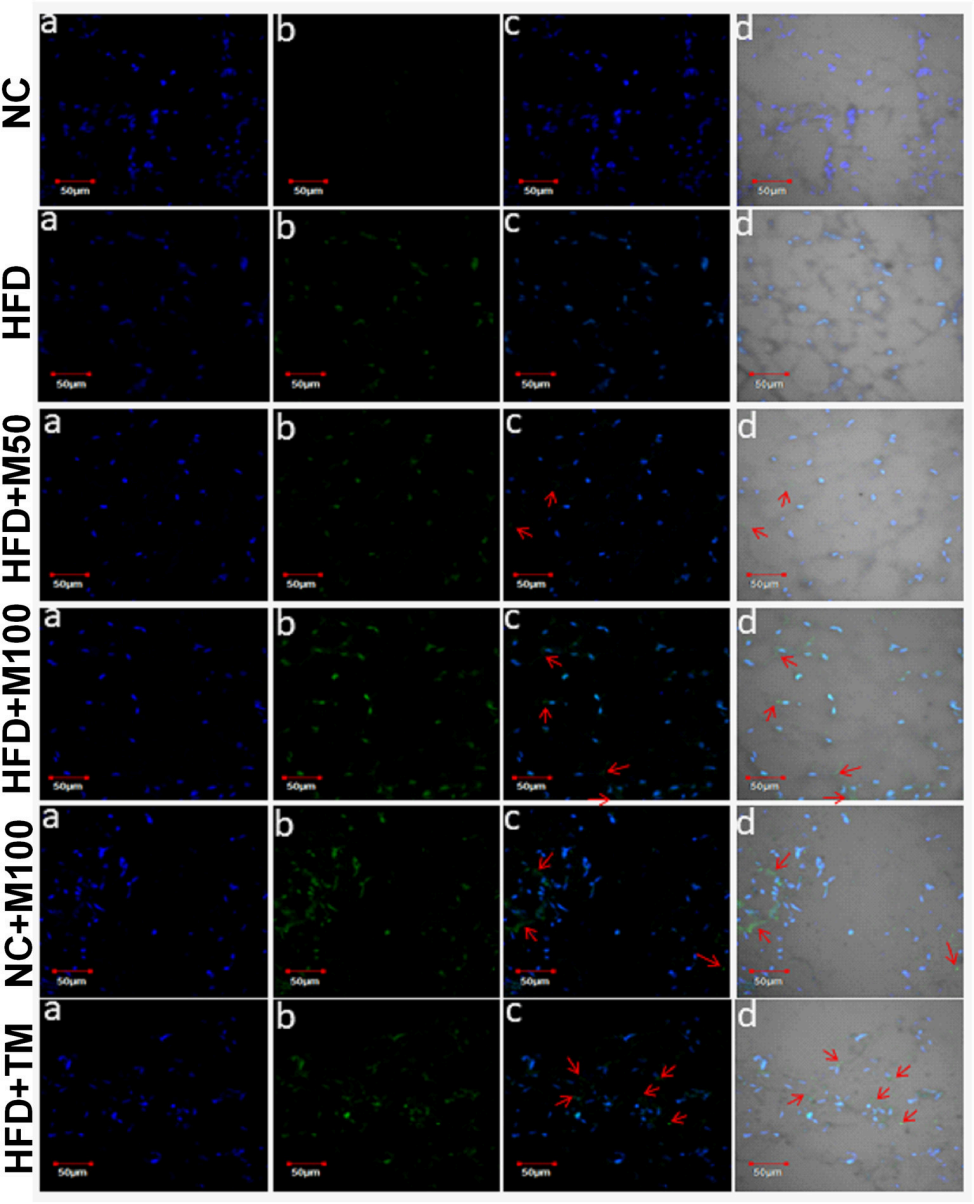
Representative images of UCP1 immunohistochemistry in adipose tissue of chronic oral and topical menthol treated HFD fed mice. **(a)** Nucleus is stained blue by DAPI; **(b)** UCP1 is stained green by FITC; **(c)** FITC and DAPI merged images; and **(d)** Merged image of DAPI, FITC with their corresponding differential interface contrast images. Arrowheads indicate immunopositive cells. NC, normal rodent chow fed; HFD, high fat diet fed; HFD+M50, HFD fed plus daily administration of 50 mg/kg menthol (*p.o*.); HFD+M100, HFD fed plus daily administration of 100 mg/kg menthol (*p.o*.); NC+M100, NC fed plus daily administration of 100 mg/kg menthol (*p.o*.); HFD+TM, HFD fed plus daily application of 10% topical menthol.

Importantly, the chronic menthol administration increased expression of *CD137* (marker of “*brite*” phenotype) in vWAT (Figure 5C) of menthol treated animals. mRNA expression of TFAM (Mitochondrial Transcription Factor A, primary transcription factor involved in energy expenditure), and IDH3a (isocitrate dehydrogenase 3a, rate limiting step of TCA cycle, and respiratory chain machinery) was significantly increased in vWAT of menthol treated animals without affecting ATP5b, and COX7a mRNA expression (Figure 5E). Moreover, mRNA expression of TFAM, IDH3a, ATP5b (ATP synthase, involved in mitochondrial energy homeostasis, and respiratory chain machinery), and COX7a (Cytochrome c oxidase polypeptide 7a, involved in respiratory chain machinery) was significantly increased in BAT of menthol treated animals (Figure 5F). Expression of UCP1 protein was found to be increased after both oral and topical menthol treatment (Figure 6).

**Figure 7:**
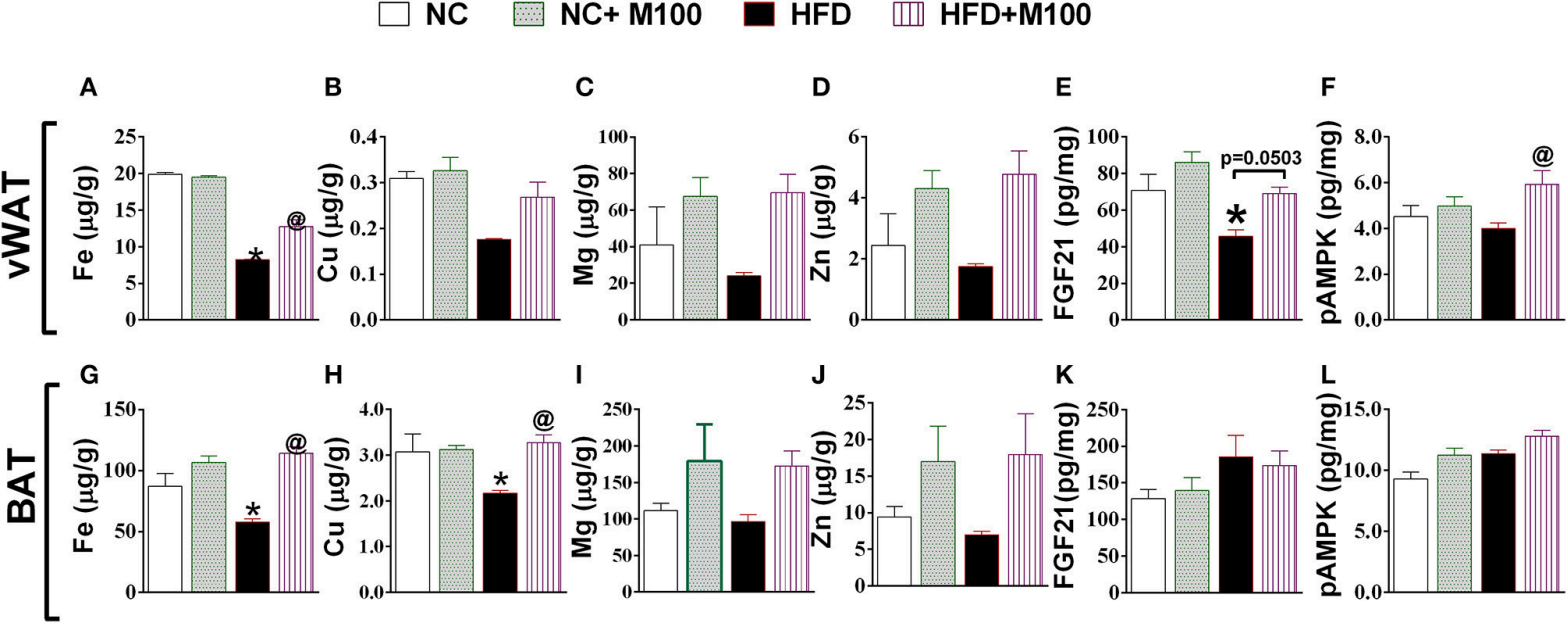
Effect of chronic oral administration of menthol on **(A–D)** metal concentration (Fe, Cu, Mg, Zn) in vWAT; **(E)** FGF-21 in vWAT; **(F)** pAMPK in vWAT; **(G–J)** Metal concentration (Fe, Cu, Mg, Zn) in BAT; **(K)** FGF-21 in BAT; **(L)** pAMPK in BAT. NC, normal rodent chow fed; HFD, high fat diet fed; HFD+M100, HFD fed plus daily administration of 100 mg/kg menthol (*p.o*.); NC+M100, NC fed plus daily administration of 100 mg/kg menthol (*p.o*.); *n* = 3; All the values are expressed as mean ± S.E.M; Analysis was carried out by one-way ANOVA followed by Tukey's multiple comparison; ^*^p < 0.05 HFD and NC+M100 vs. NC; ^@^*p* < 0.05 HFD+M100 vs. HFD fed group.

**Figure 8:**
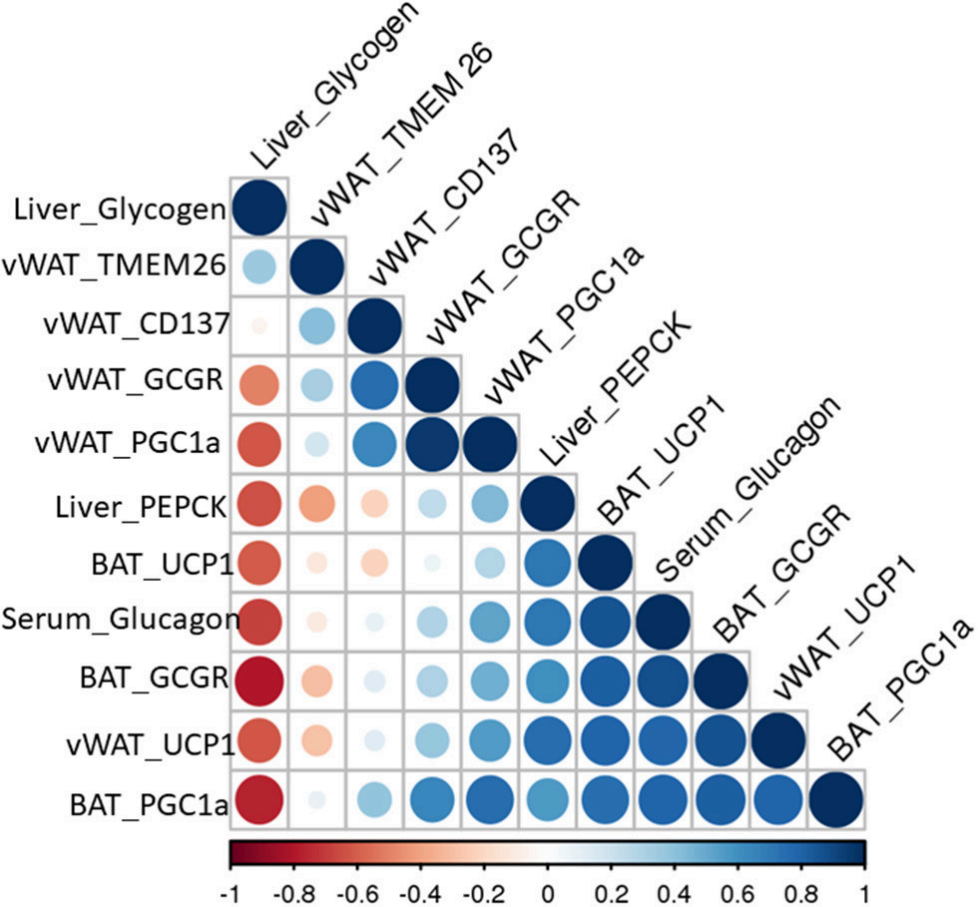
Pearson correlation matrix between glucagon mediated effects and energy expenditure related parameters observed in this study.

Fe levels were increased in vWAT and BAT of menthol treated mice (Figures 7A,E) whereas Cu levels were increased in BAT only (Figure 7F). Further, Mg and Zn levels were also increased, although non-significant, in vWAT and BAT of menthol treated mice (Figure 7). FGF21 and phosphorylated AMPK levels were also increased in vWAT of menthol treated mice (Figures 7E,F). All these changes were suggestive of “*browning*” of vWAT. High correlation was observed between glucagon dependent changes and energy expenditure markers of adipose tissue (Figure 8).

**Figure 9:**
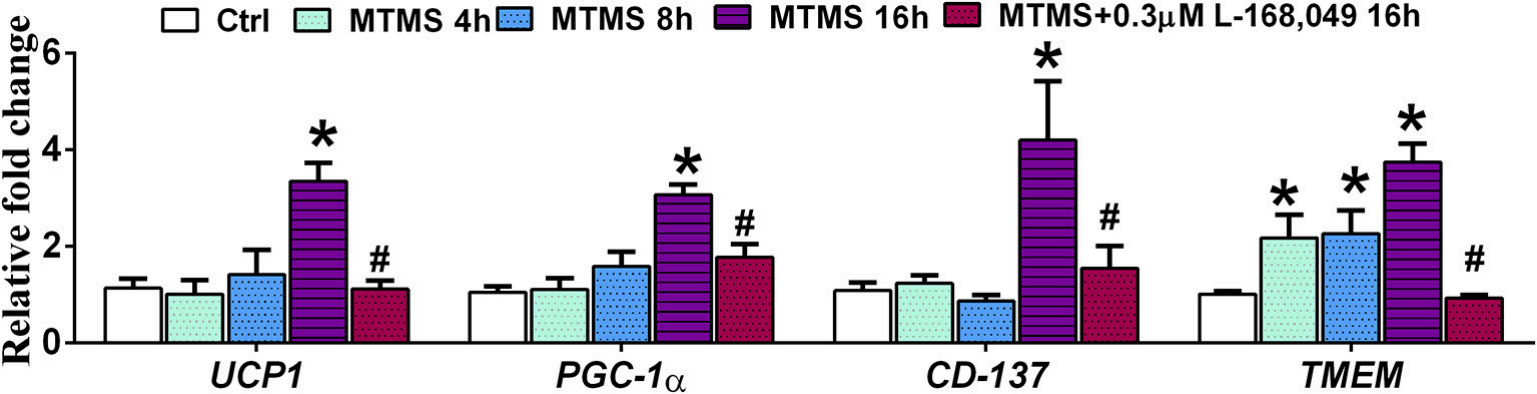
Effect of serum (glucagon containing) of menthol treated mice on mature 3T3L1 adipocytes. Effect of serum of menthol treated mouse on mRNA expression of energy expenditure related genes in mature differentiated 3T3L1 adipocytes in presence and absence of L-168,049 (non-competitive antagonist of the human glucagon receptor), *n* = 4. All the values are expressed as mean ± S.E.M; Analysis was carried out by one way ANOVA followed by Tukey's multiple comparison; ^*^p < 0.05 vs. Ctrl; #*p* < 0.05 vs. MTMS at 16 h. MTMS, menthol treated mouse serum; h, hour.

In order to study whether menthol-induced increase in serum glucagon has an ability to increase energy expenditure in adipose tissues, we replaced FBS during 3T3-L1 adipogenesis with serum of menthol treated animals (higher levels of glucagon) in maintenance media. During the adipogenesis, cells treated with serum of menthol treated animals have significantly higher expression of marker genes responsible for energy expenditure and thermogenesis as compared to untreated cells (Figure 9). Further, this increase in marker genes responsible for energy expenditure and thermogenesis was significant prevented by co-treatment with non-competitive glucagon receptor antagonist, L-168,049 (Figure 9).

## Discussion

Glucagon plays a potent role in glucose homeostasis of the body in response to hypoglycaemic conditions. In addition to hypoglycaemia, stress and cold exposure also act as stimulatory factors for glucagon secretion *in lieu* of their demand of sustained increase in energy supply during these conditions (Jones et al., 2012). The present manuscript is an attempt to understand whether activation of cold receptor, TRPM8, can pharmacologically mimic cold sensing and results in increased glucagon release and resultant enhanced energy expenditure in adipose tissues. The salient findings of present manuscript are (1) acute TRPM8 activation stimulates glucagon release and adaptive thermogenesis in mice; (2) chronic administration of menthol induces “glucagon machinery” (glucagon receptor expression, gluconeogenesis and glycogenolysis markers, FGF-21 production, and AMPK activation) in liver and adipose tissue in HFD fed animals; (3) chronic menthol administration induced serum glucagon levels are sufficient and responsible for “enhanced energy expenditure” phenotype in white adipocytes; (4) menthol administration increases iron and copper concentration in white and brown adipose tissues, hence inducing “energy expenditure and glucose utilizing metal signature;” and in last (5) menthol prevents HFD induced weight gain and related complications.

Among multiple thermoregulatory TRP channels, we have chosen TRPM8 because of two reasons: (a) TRPM8 has significant functional role in environmental temperature detection; and (b) it is non-noxious (between ~15 and 30°C) in nature (McKemy et al., 2002; Bautista et al., 2007). Sensory TRPM8 at epidermis regulates proliferation and differentiation of keratinocyte in response to cold temperatures, and regulates inflammation and other functions mediated by neuropeptides at mucosal sensory neurons of gut (Bidaux et al., 2015; De Jong et al., 2015). Its presence on skin (topical activation) and gut (oral activation) makes it suitable candidate for obesity related interventions. McCoy et al. reported that TRPM8^−/−^ exhibited prolonged hypoglycaemia in response to insulin without any impairment in glucose disposition phenomenon when challenged with glucose bolus (McCoy et al., 2013). This phenomenon can be explained hypothetically, assuming that TRPM8 is responsible for glucagon release and therefore insulin induced hypoglycaemia could not be restored in TRPM8^−/−^ mice as compared to wild type.

Acute cold exposure results in increase in oxygen consumption and serum glucagon levels while the levels of serum insulin and blood glucose remains unchanged (Seitz et al., 1981). Acute cold exposure induced increase in concentration of plasma glucagon caused rise in the concentration of hepatic cyclic AMP, thus enhancing the rate of hepatic gluconeogenesis and ketogenesis (Seitz et al., 1981). Also, during elevated glucagon levels, plasma free fatty acids were also pharmacologically elevated to ensure adequate free fatty acid substrate delivery to the liver to support hepatic ketogenesis (Seitz et al., 1981). Overall this enhanced ketogenic capacity could result due to high glucagon/insulin plasma ratio and from the energy expansive metabolic state of the liver (Foster and McGarry, 1982). Further, elevated glucagon levels are also associated with decrease in liver glycogen, increase in free fatty acids and glycerol, suggesting the enhanced glycogenolysis and lipolysis during acute cold exposure. TRPM8 agonism is mimicking the cold pharmacologically and one can expect the physiological and metabolic effects similar to cold exposure. In our study too, acute administration of TRPM8 agonists, menthol and icilin, induces increase in serum glucagon levels and adaptive thermogenesis hence corroborating well with the literature. In our chronic studies in diet-induced obesity model, we also observed significant menthol induced glucagon dependent changes, including an increase in serum glucagon/insulin ratio, decrease in liver glycogen content, decrease in liver weight, decrease in liver triglyceride levels, increase in serum glycerol content, increased expression of gluconeogenesis marker genes in liver, increase in FGF-21 and activation of AMPK. All these changes are associated with energy expenditure status of liver.

In recent years, it has been experimentally proven that non hypoglycaemic release of glucagon is associated with energy expenditure (Billington et al., 1991; Filali-Zegzouti et al., 2005; Heppner et al., 2010; Salem et al., 2016). Glucagon secretion induces net negative energy balance by both decrease in food intake and increase in energy expenditure (Jones et al., 2012; Müller et al., 2017). In the present study, there was no significant effect of menthol on feed intake over the course of 3 months (data not shown), hence the increase in energy expenditure might be an adaptive change in response to cold where heat generation is beneficial and required. Glucagon induced energy expenditure is partially independent of systemic catecholamine secretion and other pancreatic hormones (Davidson et al., 1960; Nair, 1987; Salem et al., 2016). In our study, we have seen that menthol is physiologically affecting mitochondrial activation, biogenesis and thermogenesis in adipose tissue both white as well as brown. Also, the presence of glucagon receptor and increase in the expression of genes for these receptors in both white and brown adipose tissue during menthol co-administration suggest that glucagon induced effect like increase oxygen consumption will significantly enhance energy expenditure. This can also be directly correlated with the increase in phosphorylated AMPK and FGF-21 primarily in white adipose tissue. We sought to understand that whether the menthol-induced release of systemic glucagon is responsible and sufficient to enhance energy expenditure. Serum of menthol treated animals was quantified for glucagon concentration and was used to replace normal bovine serum in mature 3T3L1 adipocytes cultures. We observed the increase in energy expenditure marker genes and “*browning*” phenotype in 3T3L1 mature adipocytes after 16-h treatment of this serum, hence suggesting that glucagon is potentially playing a role and is sufficient to induced “*browning*” machinery in adipocytes. To confirm this, we repeated the experiment in the presence of specific glucagon receptor antagonist and interestingly the effect was prevented. This substantiate that glucagon secreted during menthol treatment was sufficient and selective in inducing “*browning*” in WAT.

Recently, there is literature related to the increase in levels of iron and copper during brown adipose tissue activation but in the same study they have mentioned that there is no change in the levels of these metals in “*brite*” adipocytes (Wang et al., 2017). It has been well known that iron and copper are essential components of the mitochondrial inner membrane complexes constituting the electron transport chain, therefore, the involvement of copper and iron in energy metabolism *via* their involvement in mitochondrial function is not surprising. Our study also implied that HFD administration significantly decreased the metal concentration and the decrease was significantly prevented in the presence of menthol administration, specifically at higher doses of menthol, in WAT and BAT. We can argue that due to energy expenditure ability and prevention of lipid droplet accumulation, unit weight of adipose tissues must be having more mitochondria, hence more concentration of both metals. Our study has shown similar results in both WAT “*browning*” and BAT activation, hence are in contrast with the previous study. Also, magnesium and zinc concentration is altered during HFD administration and was significantly prevented on co-administration of menthol. Given, the role of magnesium and zinc in glucose utilization, it is not surprising that during “*browning*” or BAT activation there is significant increase in their concentrations due to high energy expenditure state. This is the first report linking these metal concentrations in tissues with HFD and “*browning*.” These have significant implications going forward as these can be common link between obesity/diabetes and micronutrient deficiencies. Although we are able to casually link the possible role of glucagon in menthol induced energy expenditure, but a clear mechanism is still lacking. Earlier studies suggest that glucagon infusion independently increased whole body energy expenditure but unable to increase BAT glucose uptake in human subjects (Salem et al., 2016). However, mice deficient in proglucagon-derived peptides (GCGKO mice) have shown that cold exposure induced brown adipose tissue energy expenditure is compromised in GCGKO mice (Kinoshita et al., 2014). These two study suggests that glucagon induced energy expenditure is an important, but not exclusive aspect of cold induced energy expenditure. Recent clinical and preclinical studies suggest that dual agonist of glucagon and GLP1 provide both glycaemic control and weight lowering effects (Henderson et al., 2016; Ambery et al., 2018). The present study cannot preclude the role of GLP1 in menthol induced prevention of weight gain and glycaemic control. Similarly, these effects might also be dependent or independent of cold induced adrenergic stimulation, which is a question of further investigation. Similarly, this study is also inconclusive to decipher sensory and/or non-sensory mediation of menthol induced glucagon increase. These studies are presently undergoing in our laboratory.

**Figure 10:**
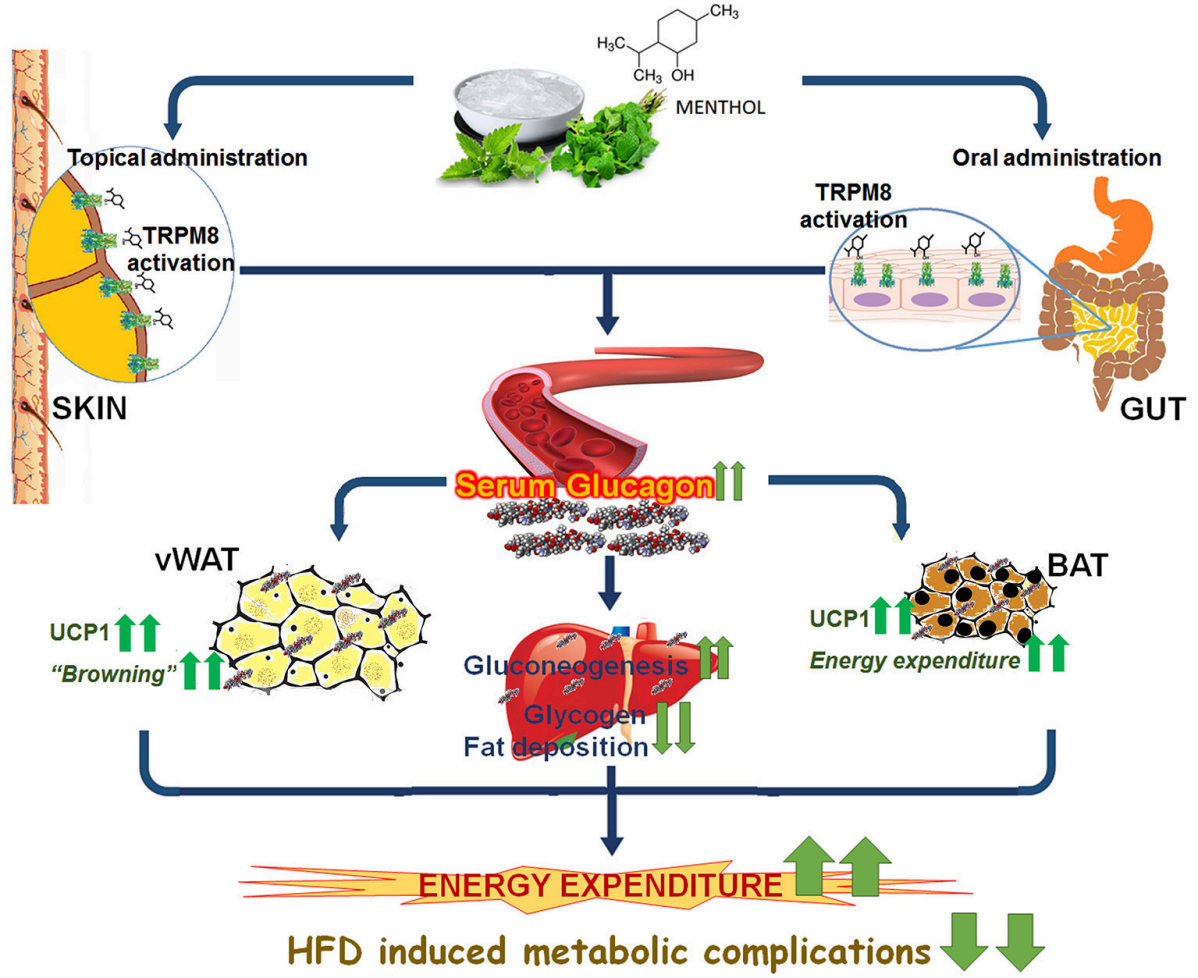
Schematic diagram presenting glucagon mediated action of TRPM8 activators in HFD fed mice. Administration/application of TRPM8 activators *via* oral/topical routes increases serum glucagon level, which leads to the activation of multiple downstream catabolic processes i.e., glycogenolysis, gluconeogenesis, browning of WAT, and activation of energy expenditure markers in WAT and BAT. These effects pharmacologically mimic cold exposure-induced phenomenon and prevent HFD-associated insulin resistance, hepatic fat deposition, and weight gain.

The present study provides evidence of TRPM8 mediated glucagon dependent mechanism of energy expenditure (Figure 10). Interestingly, the raised serum glucagon levels are sub-optimal which prevents classical glucagon mediated effects like decreased feed intake and hyperglycaemia in these animals. In conclusion we have provided evidence that TRPM8 mediated increase in serum glucagon is the signature of global shift from “fat storing state” to “fat burning state” in response to menthol administration. We believe that it is a pharmacological mimicking of cold and represents a novel strategy, which might offer another therapeutic approach to prevent and mitigate HFD induced alterations.

## Author contributions

MB, KC, and PK generated the hypothesis. MB, KC, PK, SS, and KK designed the experiments and wrote the manuscript. PK, PM, SJ, RB, VK, and DS performed the experiments, diet preparation, and data calculation. MB, KC, SS, KK, RK, SB, and RaKBo helped in manuscript writing and data presentation.

### Conflict of interest statement

The authors declare that the research was conducted in the absence of any commercial or financial relationships that could be construed as a potential conflict of interest.

## Abbreviations

AUC: Area under the concentration-time curve
BAT: Brown adipose tissue
HFD: High fat diet
ICPMS: Inductively coupled plasma mass spectrometry
FFA: Free fatty acids
NC: Normal rodent chow
UCP1: Uncoupling protein1
vWAT: Visceral white adipose tissue.
